# Economic burden of illness associated with diabetic foot ulcers in Canada

**DOI:** 10.1186/s12913-015-0687-5

**Published:** 2015-01-22

**Authors:** Robert B Hopkins, Natasha Burke, John Harlock, Jathishinie Jegathisawaran, Ron Goeree

**Affiliations:** Programs for Assessment of Technology in Health (PATH) Research Institute, St. Joseph’s Healthcare Hamilton, Suite 2000 (20th floor), 25 Main St West, Hamilton, ON Canada; Department of Clinical Epidemiology & Biostatistics, Faculty of Health Sciences, McMaster University, Hamilton, ON Canada; Division of Vascular Surgery, Faculty of Health Sciences, McMaster University, Hamilton, ON Canada

**Keywords:** Diabetes, Foot ulcers, Cost, Cost of illness, Incidence, Prevalence

## Abstract

**Background:**

The primary objective was to estimate the national burden of illness in Canada for diabetic foot ulcer (DFU) for 2011. Secondary objectives included estimating the national incidence and prevalence of DFU, and the 3-year average cost for DFU incident cases.

**Methods:**

Analyses were conducted using four national databases for the period April 1, 2006 to March 31, 2011, with cases being identified by ICD-10 CA codes. Resource utilization and costs, expressed in 2011 Canadian dollars, were estimated for DFU-related hospitalizations, emergency care (ER), same day surgeries, home care, long term care, physician visits and caregiver time losses.

**Results:**

In Canada in the year 2011, DFU was associated with 16,883 hospital admissions (327,140 days), 31,095 ER or clinic visits, 41,367 rehabilitation clinic visits, and 26,493 interventions, including 6,036 amputations and 5,796 surgical debridements. This acute institution care represented $320.5 M, and with an additional $125.4 M for home care and $63.1 M for long term care, the annual cost associated with DFU-related care was $547.0 M, or $21,371 annual cost per prevalent case. In 2011, the national prevalence of DFU was 25,597 cases (75.1 per 100,000 population), consisting of 16,161 men (63.1%) and 9,436 women (36.9%), and an estimated 14,449 incident cases. For an incident case of DFU, the average 3-year cumulative cost was $52,360.

**Conclusion:**

The annual burden for DFU cases that have at least one admission or ER/clinic visit over a 5 year period is higher than previously reported.

**Electronic supplementary material:**

The online version of this article (doi:10.1186/s12913-015-0687-5) contains supplementary material, which is available to authorized users.

## Background

Diabetes is one of the most costly and burdensome chronic diseases [1]. The economic burden of diabetes in Canada was estimated at $12.2 billion in 2010 and is expected to rise by another $4.7 billion by 2020 [2]. The direct cost of diabetes accounted for 3.5% of all public healthcare spending in Canada in the year 2005 [2].

A major concern with diabetes is the high risk of having one of many major complications such as cardiovascular, i.e., myocardial infarction, stroke, angina, or heart failure, or other types of complications such as blindness, amputation, or nephropathy [3]. Less emphasis has been placed on diabetic foot ulcers (DFU), even though they are one of the most devastating complications of diabetes [2]. Out of 2.7 million Canadians living with diabetes in 2010 [2], the probability of developing a DFU in their lifetime was 15 percent [2,4]. DFUs are caused by poor circulation associated with peripheral neuropathy and peripheral vascular disease, and if unnoticed and without early treatment, a DFU may become infected and persistent (chronic), which can lead to amputation and death [5]. Diabetic foot infections are the most common reason for admission to hospital for Canadians living with diabetes [2], and an estimated 85% of all amputations in people with diabetes are the result of a non-healing DFU [2].

In order to estimate the impact that DFU has on the health care budget and society, a current estimate of the number of new and existing cases as well as the cost per case are required. Two previous estimates were available for Canada. In the first estimate, the hospital budget cost of a DFU was $1,521 if infected and $727 if not infected. Additional costs of $17,130 for an amputation, as well as separate costs for neuropathy, prosthesis, gangrene were listed but were not pooled for an average cost per patient [6]. The second estimate which was reported as an average cost per patient included hospital admission, physician billings including procedures, but did not include supportive home care and long term care (LTC). The average cost for a patient with DFU was $2,183 in the first year, with zero care in subsequent years [7]. However, these estimates only include the cost of a single admission with a single emergency room (ER) visit, and exclude the costs of amputation, chronic care such as home care, LTC, wound or supportive dressings, and associated physician visits. In addition, the number of new and existing DFU cases has not been tracked over a multi-year period to establish prevalence and incidence.

The primary objective of this study was to estimate the national economic burden of illness in Canada for DFU for 2011. Secondary objectives included estimating the national incidence and prevalence of DFU, and the 3-year average cost for incident DFU cases.

## Methods

### Overview

First, the national rates of prevalence and incidence of DFU were estimated through an investigation of linked databases over a 5-year period. Secondly, we estimated the national resource utilization and costs for identified prevalent cases of DFU. Finally, the average 3-year cost was estimated for an incident case of DFU.

The analysis was conducted using standardized mandatory national health administrative databases for hospital admissions across Canada and available provincial data for ER and clinic visits, home care and LTC facilities. All costs were presented in 2011 Canadian dollars from a payer perspective plus additional caregiver costs. For the purpose of this research, the payer perspective includes total direct healthcare costs to public payers: hospital budget, physician fees, drugs in home care and LTC setting for infections, wounds and supportive dressings, wages for home care and LTC. The caregiver costs further included the cost of time loss for the caregiver of a patient experiencing DFU. The incident cohort analysis was identified within the province of Ontario (population 13 million, 38% of the country), primarily because of the comprehensiveness of its data coverage.

### Data sources

Four linked databases from the Canadian Institute for Health Information (CIHI) were used to gather Canadian provincial data on acute care admissions (Discharge Abstract Database – DAD) [8], emergency visits (National Ambulatory Care Reporting System - NACRS) [9], same day surgery (NACRS for Ontario, DAD for rest of Canada), home care for Ontario (Home Care Reporting System - HCRS) [10], and LTC for Ontario (Continuing Care Reporting System – CCRS) [11]. These databases were linked at the patient level to allow for the identification of unique patients, while each data source has different data elements which are briefly explained below. Ethics approval was not required to receive data from the retrospective database, consistent with CIHI policy.

To provide national estimates, 2 extrapolations were conducted due to data gaps in comprehensiveness of data within some provinces and because of the absence of data from 2 provinces. First, only Ontario had mandatory reporting in NACRS for ER and clinic visits. The extra number of cases identified in ER and clinic visits, beyond admissions alone, as well as the health care resource utilization for ER and clinic visits were both extrapolated to other provinces based on age and sex using Census data [12]. Specifically, for every age group by 10 years and by sex, we estimated the incremental cases and resource utilization that occurred beyond admission data alone. We then applied this incremental percentage to provinces without ER and clinic visit data. A second extrapolation was necessary for the absence of any data for Quebec and British Columbia (41% of country). We estimated the rate of incidence, prevalence and resource utilization by age groups of 10 years and sex that occurred in other provinces and applied these rates to the size of the population for each age group and sex cohort in Quebec and British Columbia to predict their provincial estimates.

#### Admissions

The DAD captures mandatory information on every acute care admission across Canada, and same day surgery (excluding Ontario). The data from British Columbia and Quebec were not available at the time of this research due to the provincial privacy approval process. Each patient admission has a unique record and provides ICD-10 CA codes for ‘most responsible diagnosis’, which is the diagnosis that results in the most resource intensity or contributes to the longest length of stay (LOS). In addition, up to 24 other ICD-10 codes are captured for comorbidities and secondary diagnoses, as well as up to 20 intervention codes (surgical or diagnostic).

#### Emergency room and clinic visits

The NACRS database includes 100% coverage for all of Ontario’s ER visits, hospital-based ambulatory care (i.e. same day surgery) and hospital-based clinics, such as renal dialysis or rehabilitation. Similar to DAD, the NACRS has a unique record for each patient visit and includes up to 10 diagnostic codes and 10 interventions. In addition, both DAD and NACRS provide the residency status of the patient before and after the admission, which allows tracking to home care, LTC or death.

#### Home care and long term care in Ontario

Data for home care (HCRS) and LTC (CCRS) includes assessments completed at several time points: admission, quarterly and with a change in patient’s clinical status. Both home care and LTC assessments are conducted with the InterRai Minimum Data Set (MDS) [13], which covers more than 200 questions with categories for functional limitations such as mobility status, daily activity limitations, quality of life, and wound management, etc.

### Data capture

Cases of DFU were identified using relevant ICD-10-CA codes E1^.70 and E1^.71 (further details in Additional file 1: Table S1). The data capture included all cases in Canada (with the exception of Quebec and British Columbia) that, at any time over the 5-year period (fiscal years 2006/2007 to 2010/2011 (i.e. April 1, 2006 to March 31, 2011)), had at least one ICD-10-CA diagnosis code for DFU as the most responsible diagnosis, comorbidity or secondary diagnosis for an admission, or in Ontario an ER or clinic visit. Data for home care and LTC do not include ICD codes and could not be used to identify cases with high sensitivity. After selecting all cases, all admissions/visits, home care and LTC assessments were captured for the 5-year period. The data capture does not include DFU cases that did not have an admission or ER/clinic visit over the 5 year period, such as possibly Wagner grade 1 or early grade 2.

### Prevalence and incidence

An incident case was identified as being incident in its first year that a DFU code appeared, with the two previous years not having a DFU code. The total number of unique cases by year and rates per 100,000 population were reported as prevalent cases if a DFU diagnosis code appeared during that year. As a sensitivity analysis for prevalence we included cases that had DFU-related events for up to 4 years after the first DFU code but did not have a DFU code in the subsequent years. If a subsequent DFU-related event occurred even without a DFU code the case was considered prevalent in the sensitivity analysis only.

### Resource utilization and costs of DFU

To estimate the frequency of hospitalizations, ER visits and same day surgeries attributable to DFU, the ICD-10 codes for the most responsible diagnosis were summarized for all admissions/visits by the selected cases. Then, the types of outcomes (admissions/visits) were adjudicated as related or not related, based on independent clinical expert guidance (1 wound care specialist, 1 wound care nurse) and 1 vascular surgeon (JH). For example, admissions for amputation or infection in the foot were considered related to DFU, whereas admissions for hemodialysis or cancer care were considered not related. To estimate the costs associated with hospitalizations, ER visits and same day surgeries, unit costs were applied to the resource utilization measured as relative resource intensity weight (RIW) (See Additional file 1: Table S2 for the unit costs, data sources, and main costing assumptions). The RIW for all admissions/visits are estimated by CIHI, where the value of 1.0 unit represents the average resource intensity. The national unit cost per RIW for 2011 ($5,232 per unit RIW) was multiplied to represent the costs in dollars. In addition, the RIW only captures the hospital budget portion of an admission/visit. Provincial physician billing fees that occur any time during the admission period were added to capture the cost for surgical procedures (e.g. surgeons, surgical assistants, anesthesiologists, and radiologists), diagnostic tests and daily assessments in hospital.

Admissions, ER or clinic visits that were included were ‘DFU’, ‘DFU with gangrene’, cellulitis, infection, osteomyelitis, ‘complications with procedure’, ‘complications with amputation’, rehabilitation, palliative care, convalescence, ‘peripheral angiopathy’ and ‘peripheral tissue problem’. The admissions/visits were only included if there was a DFU-related intervention (see Additional file 1: Table S4, Additional file 1: Table S5). For example, for the admissions for ‘peripheral tissue problem, 43% included an amputation and 43% included a debridement for a foot ulcer.

To estimate the costs associated with home care, the MDS InterRai assessments captured the duration of home care (time between first and last assessment), and the level of care identified by case mix group. Each assessment also enquires about health care resource utilization in the last 7 days including indication for infections, dressing changes, doctor and clinic visits, and caregiver support. For each of these cost drivers, the unit costs were applied to estimate total cost. Similar methodology was used for estimating the costs of LTC, with the only difference in methodology between home care and LTC being the different resource intensity (by case group) weighted cost per day. The costs of an antibiotic regimen and the average cost per day of dressings were obtained from the Ontario Ministry of Health and Long Term Care [14]. The cost of doctor and clinic visits were applied from the Ontario physician fee schedule, and the value of caregiver time wage loss were valued at the average national wage. To isolate the cost of DFU from other types of wound care, the costs for home care and LTC were estimated for cases that only had a stasis ulcer (which includes DFU) and excluded cases that required wound care for pressure ulcers and other types of skin conditions (e.g. burns, cutaneous lesions other than ulcers, skin tears or cuts, surgical wounds, corns/callouses/structural/fungi).

### Incident cohort analysis

The incident cohort for FY2008/2009 was followed for a total of 3 years (incident year plus 2 subsequent years). For each of the years, the rates of admissions, visits and procedures were captured. In addition, the proportion of the incident cohort that required the use of home care, the use of wound care in LTC by existing long term residents, and the number of cases that transitioned into LTC were captured. The cost per year was estimated and was reported as a 3-year cumulative cost.

## Results

### Data capture and identification of cases

The cases that reported an ICD-10 code for ‘DFU’ or ‘DFU with gangrene’ as most responsible diagnosis represented 60% of cases identified. The remaining 40% of cases were identified with DFU as a comorbidity. In Ontario, 12% of cases were identified only with ER and clinic visits. For provinces that did not have ER and clinic visit databases, results were increased by 12% to account for these incremental cases. From these estimates, the cases in Quebec and British Columbia were then extrapolated based on estimates from the rest of the country.

### Prevalence and incidence

The estimated national number of prevalent cases increased from 19,740 in 2007 to 25,597 in the year 2011, which represents an increase of 29.7% (7.4% per year) over the 5-year period. The prevalent rate for Canada in 2011 was 75.1 per 100,000 general population (0.075%). The most common age group for cases was 55 to 64 years, while the highest prevalent rate was in the 75 to 84 years age group (Table 1). The cases were 63% men (95.5 per 100,000) and 37% women (54.9 per 100,000). The rate varied from 64.7 in Ontario to 106.2 in Manitoba. Most provinces reported a rate of 70 to 90 per 100,000 population (Additional file 1: Table S3).

**Table 1:** **Prevalent and incident cases based on DFU code, by age group and sex, Canada, 2011**

**Age group**	**Prevalent cases (rates per 100,000)**			**Incident cases (rates per 100,000)**		
	**Women**	**Men**	**All**	**Women**	**Men**	**All**
<35 years	278 (3.8)	357 (4.7)	635 (4.3)	164 (2.2)	197 (2.6)	361 (2.4)
35 to 44 years	606 (25.9)	1,065 (44.9)	1,671 (35.4)	314 (13.4)	524 (22.1)	838 (17.8)
45 to 54 years	1,383 (51.2)	2,738 (101.0)	4,121 (76.1)	738 (27.3)	1,380 (50.9)	2,118 (39.1)
55 to 64 years	2,180 (101.1)	4,478 (213.9)	6,666 (156.6)	1,171 (54.1)	2,277 (108.8)	3,448 (81.0)
65 to 74 years	2,097 (156.3)	4,060 (329.5)	6,157 (239.2)	1,252 (93.3)	2,298 (186.5)	3,550 (137.9)
75 to 84 years	2,031 (224.5)	2,861 (411.1)	4,892 (305.7)	1,317 (145.6)	1,775 (255.0)	3,092 (193.2)
85+ years	853 (198.2)	602 (288.5)	1,455 (227.7)	626 (145.4)	416 (199.4)	1,042 (163.0)
**Total**	**9,436** (**54.9**)	**16,161** (**95.5**)	**25,597** (**75.1**)	**5,582** (**32.5**)	**8,867** (**52.4**)	**14,449** (**42.4**)

DFU: Diabetic Foot Ulcer.

The national incidence of DFU was 14,449 cases for both women and men in 2011 (Table 1). Most of the cases were men (61%), while the rate per 100,000 population was 32.5 for women and 52.4 for men, with age distributions similar to prevalent cases. The rates of incidence ranged from 36.9 in Ontario to 57.2 in Manitoba, while most provinces varied from 40 to 60 per 100,000 population (Additional file 1: Table S3).

In a sensitivity analysis on the estimate of prevalence, 34% had an admission, ER, clinic visit, or a procedure that was consistent with DFU in the year after the incident index year, despite not having a DFU code in these subsequent years. In the third year, 24% still had care relevant to DFU, while in the fourth year 21% still had relevant care, regardless of a specific DFU code. Based on having relevant care after a DFU code, there were 3,989 additional cases in the year 2011, 15.6% higher than with DFU code alone. Nationally, that translates into 29,586 prevalent cases of DFU instead of 25,597.

### Resource utilization and costs of DFU

The number of admissions related to DFU in 2011 was 16,863 (Table 2). Men accounted for 67% of the admissions, and the average number of admissions per prevalent case was 0.66 (SD = 0.98, median = 1, min = 0, max = 9). The most common admissions were for ‘peripheral angiopathy’ (25%) or admissions coded as ‘DFU’ (18%) or ‘DFU with gangrene’ (17%). The 16,863 admissions in 2011 represent 327,140 hospital bed days.

**Table 2:** **Admissions and emergency/clinic visits related to DFU, by sex, Canada, 2011**

**Most responsible diagnosis**	**Admissions**			**ER and clinic visits**		
	**Women**	**Men**	**All**	**Women**	**Men**	**All**
DFU	881	2,134	3,015	2,827	6,380	9,207
DFU with gangrene	832	2,051	2,883	490	1,110	1,600
Infection/ sepsis	265	405	670	264	532	796
Cellulitis lower limb	744	1,103	1,847	3,630	6,277	9,907
Ulcer lower limb	160	209	369	101	187	288
Osteomyelitis	302	627	929	253	1,035	1,288
Complications with procedures	117	293	410	437	882	1,319
Complications with amputations	133	345	478	60	234	294
Surgical dressing care				1309	2220	3,529
Rehabilitation	195	435	630	11,435	29,932	41,367
Palliative care	61	73	134	0	30	30
Convalescence	209	484	693	3	9	12
Peripheral angiopathy	1,462	2,772	4,234	742	1,486	2,228
Peripheral tissue problems	202	369	571	179	418	597
**Total**	**5,563**	**11,300**	**16,863**	**21,730**	**50,732**	**72,462**
Total (less rehabilitation clinic)				10,295	20,800	31,095

DFU: Diabetic Foot Ulcer, ER: Emergency Room.

The estimated number of ER or clinic visits related to DFU was 72,462 in 2011, which included 41,367 rehabilitation clinic visits and 31,095 non-rehab ER visits (Table 2). This represents an average of 2.83 combined annual ER or clinic visits, of which on average 1.62 (SD = 8.01, median = 1, min = 0, max = 144) were rehabilitation and 1.21 (SD = 2.42, median = 1, min = 0, max = 71) were non-rehabilitation. Excluding rehabilitation, the most common non-rehabilitation ER visits were ‘cellulitis’ (32%) and ‘DFU’ (30%).

The estimated number of interventions associated with DFU was 26,493 in the year 2011, with 69% performed in men. The interventions included 6,036 unique cases with amputation (24% of interventions), 5,796 with surgical debridement (24%) and 6,663 with antibiotics (intravenous or injectable) (26%), 1,550 angioplasty of the leg (6%), and 1,033 femoropopliteal bypass of the leg (4%) (Additional file 1: Table S6). Of the 6,036 cases of amputation, 2,051 were tarsal, metatarsal, or ankle (fore, mid and hind foot), and 2,011 were below the knee (tibia-fibula). (Average costs per admission, emergency and clinic visits, and interventions are provided in Additional file 1: Table S7, Additional file 1: Table S8).

The rate of admissions varied across the provinces by +/− 11% for women and +/−8% for men. Rates of hospitalizations were higher in the prairie provinces (Manitoba and Saskatchewan) and lower in the maritime provinces (New Brunswick, Nova Scotia, Prince Edward Island, and Newfoundland and Labrador).

In 2011 the resource utilization associated with DFU for acute institutional care was $358.6 M, of which $320.5 M was for admissions (89%), $19.1 M for ER and clinic visits (5%), and an additional $19.0 M for interventions (5%) (Table 3). If we had included admissions/visits that did not have a DFU-related intervention, the cost of acute care would be 17% higher.

**Table 3:** **Summary of burden of illness of DFU in Canada in 2011, including prevalence, incidence, resource utilization and costs**

**Epidemiology:**	**n**
Prevalent cases	25,597
Prevalence rates per 100,000	75.1
Incident cases	14,449
Incidence rates per 100,000	42.4
**Resource utilization:**	**n**
Admissions	16,863
ER/Clinic visits	72,462
Interventions	26,493
*Amputations*	*6,036*
**Cost:**	**$ millions (2011 CAD$)**
Total acute institutional care	$358.6
*Admissions*	*$320.5*
*ER/Clinic visits*	*$19.1*
*Interventions*	*$19.0*
Home care	$125.4
Long term care – current residents	$51.7
Long term care – new residents	$11.4
**Total cost**	**$547.0**
**Average cost per prevalent case**	**$21,371**

#### Resource utilization and cost of home care and LTC

There were 1,808 DFU cases with repeated home care assessment that did not include pressure ulcers or other skin conditions, with a mean duration of home care of 238 days (average age of 70 years; 62% male). The average cost per home care case was $9,934, which consisted of $3,948 for dressings, $38 for oral and IV antibiotics, $739 for physician and clinic visits, and $5,209 for the home care (wages and benefits of staff). In addition, the dollar value of time loss for a caregiver was on average $12,432, resulting in a total home care cost of $22,366 per case with DFU.

There were 461 DFU cases that had repeated assessments for LTC that did not include pressure ulcers and other skin conditions. The average age was 78 years, 49% men, and the average duration of LTC was 110 days. The average costs included $2,090 for wound or supportive dressings, $65 for oral and IV antibiotics, $340 for physician or clinic visits, and institutional costs. For cases that were already residing in LTC, the incremental institutional cost over the pre-existing care was $7,917, while the cost of institutional care that was fully attributable to DFU (with zero pre-existing care) was $18,530. This resulted in the total cost of DFU in existing LTC residents of $10,413, while the full attribution costs for new admissions to LTC was $21,025, under the assumption of no time loss for caregivers.

Nationally, from the records for admissions/visits, there were 21.9% cases that indicated the use of home care, and a separate 13.9% had a residency status of LTC, of which 4.3% were existing LTC residents, and 9.6% were new to LTC. Applying the average costs of home care and LTC, this represents an additional $125.4 M for home care, $51.7 M for existing LTC residents, and $11.4 M for new LTC residents, for a total annual cost of $547.0 M for DFU in Canada (Table 3). Based on a prevalence of 25,597 cases in the year 2011, this represents $21,371 per prevalent case.

### Incident cohort analysis

5,015 cases in Ontario were identified that were incident in FY2008/2009 with their care followed for FY2008/2009, FY2009/2010 and FY2010/2011. The average age was 68 years (SD = 14, min = 19, max = 102) and 60% were men (Additional file 1: Table S9). The most common comorbidities were diseases of the arteries and capillaries (29%), renal failure (25%), ischemic heart disease (22%), and heart failure (20%). The cohort was followed for up to three years, however, 1,325 deaths occurred during this time for a Kaplan Meier estimate of mortality of 26.4%.

Of the 5,015 incident cases, with all cases identified by having an admission/visit in the first year, 59.3% had admissions for any reason in the second year (33% relevant) and 47.3% had admissions/visits for any reason in the third year (22% relevant) (Additional file 1: Table S10). Amputations occurred in 17.6% of the cases in the first year, 7.3% in second year, and 3.7% in third year (Additional file 1: Table S11). The acute care cost was $13,031 in the first year, $5,314 in year 2 and $3,040 in year 3 (Table 4). Overall, home care was the largest contributor to 3-year costs ($20,180 or 39%) followed by acute care admissions ($18,957 or 36%). The 3-year average cost of an incident case was $52,360, of which $26,380 (49%) occurred in year 1, $15,063 (28%) in year 2, and $11,544 (22%) in year 3.

**Table 4:** **Average 3-year cumulative cost for incident DFU cases, by year, 2009–2011 (2011 CAD$)**

**Type of care**	**Year 1**		**Year 2**		**Year 3**		**3-year cost**
**Acute institutional care**							
Admissions	$11,492		$4,766		$2,699		$18,957
ER visits	$370		$122		$88		$580
Procedures	$746		$321		$154		$1,221
**Subtotal – acute**	**$13,031**		**$5,314**		**$3,040**		**$20,758**
**Non-acute care by residency status, (%)**							
At Home (no LTC, no home care)	$0	(32.9%)	$0	(49.3%)	$0	(55.8%)	$0
New to long-term care	$3,568	(17.0%)	$2,505	(11.9%)	$1,761	(8.4%)	$7,835
Resides in long-term care	$1,245	(12.0%)	$1,237	(11.9%)	$1,104	(10.6%)	$3,587
Used home care:	$8,535	(38.2%)	$6,006	(26.9%)	$5,639	(25.2%)	$20,180
Home care - direct medical	$3,791		$2,668		$2,505		$8,964
informal caregiver	$4,744		$3,338		$3,134		$11,217
**Subtotal - non-acute**	**$13,349**	**(100%)**	**$9,749**	**(100%)**	**$8,504**	**(100%)**	**$31,602**
**Total cost**	**$26,380**		**$15,063**		**$11,544**		**$52,360**
*Total Cost - direct medical*	$21,212		$11,619		$8,312		$41,143
*Total Cost - informal care*	$4,744		$3,338		$3,134		$11,217

Index year for incidence cohort was 2009, with previous 2 years without DFU diagnosis in any record.

Costs exclude outpatient billings for non-home care and non-LTC cases.

59% of cohort had year 2 costs, 47% of cohort had year 3 costs.

## Discussion

In 2011, the estimated number of prevalent cases of DFU in Canada was 25,597 rising over the 5 year period at a rate of 7.4% growth per year. The cost associated with each new DFU case over the first 3 years for a case that had an admission or ER/clinic visit was $52,360, which includes admissions, ER and clinic visits, drugs, wounds and supportive dressings, nursing-provided home care, LTC and caregiver time loss. In 2011, DFU was associated with 16,863 admissions, resulting in 327,140 hospital bed days. This translates into a rate of hospitalization for DFU of 88 per 100,000 adults, which is similar to the rate of admissions for stroke of 124 per 100,000 adults [15].

While the use of national administrative databases have allowed us to capture many DFU cases, the true number of cases may be higher due to the limitations around DFU coding in the current databases for follow-up care. Even though the care received beyond 3 years post-initial diagnosis may be DFU-related, such as having dressing changes or amputations, cases without a DFU code were not included in the estimates. By including the percentage of cases that was considered to have received relevant DFU care, the true prevalence was estimated to be 15.6% higher. In addition, the true prevalence may even be higher if we could include less severe diabetic foot wounds (i.e. Wagner Grade 1 or non-urgent cases of Wagner Grade 2) that were under physician care and did not have a recent admission or an ER visit. Similarly, access to hospital-based wound care clinics was gathered through the NACRS database, however 39% of wound care teams across Ontario are not located within out-patient departments associated with hospitals [16]. Our estimates of rates of prevalence and incidence are reported relative to the general population. If we had recent rates of prevalence of diabetes, we could estimate the rates of DFU relative to the diabetic population, as well as providing a lifetime risk of DFU using cross-sectional methods.

In recent years, there may have been factors that contributed to increased cost per patient and total cost. There have been additions to the treatment options for patients with DFU beyond inflation for the average cost of health care, including new bioactive gauzes, and an increased focus to increase the blood flow to the foot with peripheral vascular surgery in order to save the limb to prevent amputation. Both of these factors may have contributed to increased intensity of DFU care, while the factors may also have increased the duration of care for DFU. In addition to more treatment options, there has been an increased focus to intervene earlier in the progression of DFU by treating Wager grade 1 or 2 wounds. Also, there is a high rate of mortality for patients with DFU which suggests there would be increased resource intensity with severe cases to prevent death.

In addition to capturing most cases of DFU, multi-faceted costs of DFU-related care were identified over many years, such as osteomyelitis, angiopathy, amputation and complications. Based on our analysis, 22% of incident cases had a DFU-related admission or ER visit at 3 years after the first admission. Estimates from previous work emphasized DFU without an amputation, or focused on amputations only. For example, for the first year of DFU without an amputation, based on literature values from the United States in 2013, the cost of DFU care was $2,147, and a separate cost was $9,041 if an amputation was involved [17]. For Medicare patients in the United States, the 12-month cost for DFU that excluded drug costs and caregiver or patient work loss was $11,296 [18]. A literature review for international estimates was reported in the Lancet in 2005 and the only evidence that was available at the time was from small case series [19]. Ontario multi-year period estimates that have focused on amputation including admissions, ER visits and drugs, but excluding home care and LTC, provided estimates of $34,470 for the first year of an amputation and $4,721 for all subsequent years [20]. Based on this project’s estimates, the added costs of home care and LTC represent an additional 41%.

This analysis included several key components (e.g., multi-year patient-level data in many administrative datasets; national and provincial data), however several limitations are to be noted. First, the number of cases was based on two extrapolations: the added cases that arise from ER and clinic visits, and the extrapolation to the provinces with missing data (i.e. British Columbia and Quebec accounting for 40% of the national population). Based on the variation that exists across provinces, we might expect that the extrapolated costs to the missing provinces could be different by +/− 11% for women and +/− 8% for men. Second, although the most responsible diagnosis was used at discharge to identify the study population, some of the days spent in hospitals may be have been related to other conditions. Similarly, for the home care and LTC cost per incident cases were used that had only DFU and cases with pressure ulcers were excluded to derive an average cost for health care. As such, it may be that the cost to treat DFU ulcers is higher due to increased comorbidity, or lower due to coupled care. Similarly, our costs are based on a unique patient, and each patient may have experienced multiple DFU. A third limitation is the percentage of the cost of admissions/visits and the number of admissions/visits that is attributed to DFU. We acknowledge that the presence of other comorbidities will increase the cost for each episode and increase the probability of an admission/visit. In the health economics literature, 3 methods have been proposed to account for attribution bias. The first is selective attribution, which is to be parsimonious in choosing health care that is disease-related, which is the approach we have taken here by requiring an admission to have an associated intervention. The second method is to conduct a pre-post analysis to estimate the annual cost of patients with diabetes before and after a DFU diagnosis. A third and more common method is to use matching or regression methods where patients with diabetes are stratified by presence or absence of DFU. The difference between the annual cost of the patients is then fully attributed to DFU. Both of these latter 2 methods, as well as any regression adjustment should include a larger dataset than we obtained. Future work should include estimating the attribution percentage from using either method, and to explain factors that contribute to variation in costs.

## Conclusion

In conclusion, the burden associated with DFU in Canada in 2011 was estimated to be $547.0 M. As shown in this sensitivity analysis, when including cases that had DFU-related care, (despite not having a DFU code), the annual cost might be 16% higher, and the cost of acute care could be 17% higher if we included admissions without disease-related interventions. Since our trend over 5 years was a 7.4% increase in the rate of prevalence, the cost of DFU could increase in the future. As such, improved management of diabetes, prevention and early treatment of DFUs are essential to decrease the human and economic burden of DFU. In 2012 The Council of the Federation, the national and provincial premiers in Canada, recommended that the healthcare systems prioritize interventions that slow or halt the progression of diabetes complications [21].

## Additional file

Additional file 1: Table S1. Identification of diabetic foot ulcers in CIHI data (ICD-10-CA). **Table S2.** Unit costs, data sources and main costing assumptions. **Table S3.** Prevalent and incident cases based on DFU code, by province and sex, Canada, 2011. **Table S4.** Frequency of interventions associated with different types of admissions, Ontario, 2007-2011. **Table S5.** Frequency of interventions associated with different types of ER and clinic visits, day procedures, Ontario, 2007-2011. **Table S6.** Interventions related to DFU code, by sex, Canada, 2011. **Table S7.** Average length of stay and costs for admission and ER/clinic visits related to DFU, Canada, 2011 (2011 CAD$). **Table S8.** Physician fees for interventions, Ontario, (2011 CAD$). **Table S9.** Baseline demographics of incident DFU cohort. **Table S10.** Incident based analysis, summary of reasons for admission or ER/clinic visit, by year, 2009-2011. **Table S11.** Incident based analysis, types of amputations, by year, 2009-2011.

## References

[1] Lipscombe LL, Hux JE (2007). Trends in diabetes prevalence, incidence, and mortality in Ontario, Canada 1995–2005: a population-based study. Lancet.

[2] Canadian Association of Wound Care (2013). Statistics.

[3] Goeree R, Lim ME, Hopkins R, Blackhouse G, Tarride JE, Xie F, O’Reilly D (2009). Prevalence, total and excess costs of diabetes and related complications in Ontario, Canada. Can J Diabetes.

[4] American Diabetes Association (1999). Consensus development conference on diabetic foot wound care. Diabetes Care.

[5] Shannon RJ (2007). A cost-utility evaluation of best practice implementation of leg and foot ulcer care in the Ontario community. Wound Care Canada.

[6] Ray JA, Valentine WJ, Secnik K, Oglesby AK, Cordony A, Gordois A, Davey P, Palmer AJ (2005). Review of the cost of diabetes complications in Australia, Canada, France, Germany, Italy and Spain. Curr Med Res Opin.

[7] O’Brien JA, Patrick AR, Caro JJ (2003). Cost of managing complications resulting from type 2 diabetes mellitus in Canada. BMC Health Serv Res.

[8] Canadian Institute for Health Information (2011). Data Quality Documentation for External Users: Discharge Abstract Database (DAD), 2010–2011.

[9] Canadian Institute for Health Information (2012). Data Quality Documentation for External Users: National Ambulatory Care Reporting System (NACRS), 2010–2011.

[10] Canadian Institute for Health Information (2012). Data Quality Documentation for External Users: Home Care Reporitng System (HCRS), 2010–2011.

[11] Canadian Institute for Health Information (2012). Data Quality Documentation for External Users: Continuing Care Reporting System (CCRS), 2010–2011.

[12] Statistics Canada (2012). Estimates of Population, By Age Group And Sex For July 1, Canada, Provinces And Territories, Annual. Table 051–0001.

[13] Poss J, Murphy KM, Woodbury MG, Orsted H, Stevenson K, Williams G, Macalpine S, Curtin-Telegdi N, Hirdes JP (2010). Development of the interRAI Pressure Ulcer Risk Scale (PURS) for use in long-term care and home care settings. BMC Geriatr.

[14] Health Quality Ontario (2014). Hyperbaric Oxygen Treatment for Diabetic Ulcers. Publications and OHTAC Recommendations: Work in Progress. Ontario Ministry of Health and Long Term Care.

[15] Canadian Institute for Health Information (2012). Health Indicators.

[16] Trubiani G, Pham B, Stern A, Carcone S, Rosen L, Krahn M (2011). Specialized Multidisciplinary Community-Based Care for Chronic Wounds: A Field Evaluation. Toronto Health Economics and Technology Assessment Collaborative (THETA).

[17] Alvarez P, Ward A, Chow W, Vo L, Martin S (2013). Direct Medical costs of Diabetic Complications in the United States. Value in Health.

[18] Rice JB, Desai U, Cummings AK, Birnbaum HG, Skornicki M, Parsons N (2013). Medical, Drug, and Work-Loss Costs of Diabetic Foot Ulcers. Value in Health.

[19] Boulton AJ, Vileikyte L, Ragnarson-Tennvall G, Apelqvist J (2005). The global burden of diabetic foot disease. Lancet.

[20] O’Reilly D, Hopkins R, Blackhouse G, Clarke P, Hux J, Tarride JE, Dolovich L, Goeree R (2007). Long-term cost-utility analysis of a multidisciplinary primary care diabetes management program in Ontario. Can J Diabetes.

[21] The Council of the Federation (2012). From Innovation to Action: The First Report of the Health Care Innovation Working Group. The Council of the Federation.

