# Supplementary material for: Economic burden of illness associated with diabetic foot ulcers in Canada
Source: BMC Health Serv Res. 2015 Jan 22;15:13. doi: 10.1186/s12913-015-0687-5 (PMC4307900; doi:10.1186/s12913-015-0687-5)
Supplement: Additional file 1: Table S1. — Identification of diabetic foot ulcers in CIHI data (ICD-10-CA). Table S2. Unit costs, data sources and main costing assumptions. Table S3. Prevalent and incident cases based on DFU code, by province and sex, Canada, 2011. Table S4. Frequency of interventions associated with different types of admissions, Ontario, 2007-2011. Table S5. Frequency of interventions associated with different types of ER and clinic visits, day procedures, Ontario, 2007-2011. Table S6. Interventions related to DFU code, by sex, Canada, 2011. Table S7. Average length of stay and costs for admission and ER/clinic visits related to DFU, Canada, 2011 (2011 CAD$). Table S8. Physician fees for interventions, Ontario, (2011 CAD$). Table S9. Baseline demographics of incident DFU cohort. Table S10. Incident based analysis, summary of reasons for admission or ER/clinic visit, by year, 2009-2011. Table S11. Incident based analysis, types of amputations, by year, 2009-2011. [file 12913_2015_687_MOESM1_ESM.docx]

**Additional file 1: Table S1: Identification of diabetic foot ulcers in CIHI data (ICD-10-CA)**

| **Condition of Interest** | **ICD-10-CA Code v2009**  **(2009/10 to 2010/11)** | **ICD-10-CA Code v2006**  **(for years 2006/07 to 2008/09)** |
| --- | --- | --- |
| Diabetic foot ulcer  Includes:   - Chronic foot ulcer - Decubitus ulcer | E1^.70 Diabetes mellitus with foot ulcer (angiopathic) (neuropathic) | E1^.70 Diabetes mellitus with ulcer (of lower limb) (angiopathic) (neuropathic)  +  L97.- Chronic skin ulcer of lower limb (leg and foot) |
| Diabetic foot ulcer with gangrene  Includes:   - Chronic foot ulcer with gangrene - Decubitus ulcer with gangrene | E1^.71 Diabetes mellitus with foot ulcer (angiopathic) (neuropathic) with gangrene | E1^.71 Diabetes mellitus with ulcer (of lower limb) (angiopathic) (neuropathic) with gangrene  +  L97.- Chronic skin ulcer of lower limb (leg and foot) |
| Diabetic ulcer of lower limb, not involving foot | E1^.68 Diabetes mellitus with other specified complication, not elsewhere classified  +  L97 Chronic skin ulcer of lower limb (not foot), or  L98.4 Chronic ulcer of skin, NOS (not lower limb), or  L89.- Decubitus [pressure] ulcer and pressure area | E1^.9 Diabetes mellitus without (mention of) complications  +  L98.4 Chronic ulcer of skin, NOS (not lower limb), or  L89.- Decubitus [pressure] ulcer and pressure area |

The diagnosis code was required to appear in at least one of the years (FY2006/07 to FY2010/11). The diagnosis code could be any of: most responsible diagnosis; secondary diagnosis; or pre-admit or post-admit comorbidity.

Notations: ICD: International Classification of Diseases. CIHI: Canadian Institute for Health Information. CA: Canadian. NOS: Not otherwise specified

**Additional file 1: Table S2: Unit costs, data sources and main costing assumptions**

| **Cost component** | **Item** | **Unit costs (*data source*)** | **Main costing assumptions** |
| --- | --- | --- | --- |
| Acute Care  (includes admissions, emergency, day surgery with identical methodology) | Cost per RIW | $5,232 (*CIHI*) | - Quebec hospitalizations extrapolated from all other Canadian provinces - Ontario data on number of same day surgeries and emergency room visits extrapolated to Canada - Patient-level costing |
|  | Physician visit fees* | *$77.20* (admission);  *$58.80* (2^nd^ , 3^rd^ and last day);  *$31.00* (other days)  *(OSBPS)* |  |
|  | Surgeon, surgical assistant and anesthesiologist procedure fees for assessment, procedure and follow-up | Range from $647 for surgical debridement, to $2,067 for above knee amputation *(OSBPS*) |  |
| Outpatient physician services | Physician visit fees* | General practice: Consultation $61.55  (OSBPS) | Assume consultation fee for each visit |
| Caregiver wage losses | Cost per day | $24.12 per hour x 8 hours per day *(Statistics Canada)* | Number of days based on Ontario assessments in home care |
| Home care | Cost per resource intensity by case group, additional costs for antibiotics, dressings, clinic visits | Average $152.57 per week  adjusted for case group intensity (MDS Inter-rai) | - Ontario data on number of recipients extrapolated to Canada - Length of stay based on average Ontario data and unit costs from Ontario |
| Long-term care | Cost per resource intensity by case group | $152.94 per day  (adjusted for case group intensity) (MDS Inter-rai) | For new and existing cases in LTC. Length of care based on Ontario assessments, unit costs from Ontario |

*fees associated with surgeons, anesthesiologists, radiologists are not included in RIW

Notations: RIW: resource intensity weight; CIHI: Canadian Institute for Health Information; OSBPS: Schedule of Benefits, Physician Services, Ontario; MDS Inter-rai: Minimal data set

**Additional file 1: Table S3: Prevalent and incident cases based on DFU code, by province and sex, Canada, 2011**

| **Province/**  **Territory** | **Prevalent Cases (rates per 100,000)** | | | **Incident Cases (rates per 100,000)** | | |
| --- | --- | --- | --- | --- | --- | --- |
|  | **Women** | **Men** | **All** | **Women** | **Men** | **All** |
| NFLD LAB | 181 (69.7) | 273 (109.2) | 454 (89.1) | 109 (42.0) | 152 (60.8) | 261 (51.2) |
| Nova Scotia | 301 (62.1) | 566 (123.7) | 867 (92.0) | 187 (38.6) | 320 (70.0) | 507 (53.8) |
| PEI | 38 (52.3) | 79 (113.6) | 117 (82.3) | 26 (35.8) | 39 (56.1) | 65 (45.7) |
| New Brunswick | 260 (67.9) | 456 (123.6) | 716 (95.3) | 163 (42.6) | 232 (62.9) | 395 (52.6) |
| Quebec | 2,330 (58.5) | 3,935 (100.4) | 6,265 (79.2) | 1,381 (34.7) | 2,159 (55.1) | 3,540 (44.8) |
| Ontario | 3,172 (47.4) | 5,380 (82.6) | 8,552 (64.7) | 1,874 (28.0) | 3,001 (46.1) | 4,875 (36.9) |
| Manitoba | 492 (79.3) | 820 (133.3) | 1,312 (106.2) | 273 (44.0) | 434 (70.6) | 707 (57.2) |
| Saskatchewan | 314 (59.8) | 599 (115.1) | 913 (87.3) | 196 (37.3) | 330 (63.4) | 526 (50.3) |
| Alberta | 1,047 (57.5) | 1,768 (93.0) | 2,815 (75.7) | 603 (33.1) | 937 (49.3) | 1,540 (41.4) |
| British Columbia | 1,292 (56.6) | 2,268 (101.0) | 3,560 (78.6) | 765 (33.5) | 1,251 (55.7) | 2,016 (44.5) |
| Territories | 9 (16.6) | 17 (29.6) | 26 (23.3) | 5 (9.2) | 12 (20.9) | 17 (15.2) |
| **Canada** | **9,436 (54.9)** | **16,161 (95.5)** | **25,597 (75.1)** | **5,582 (32.5)** | **8,867 (52.4)** | **14,449 (42.4)** |

PEI: Prince Edward Island. NFLD LAB: Newfoundland and Labrador. DFU: Diabetic Foot Ulcer

**Additional file 1: Table S4: Frequency of interventions associated with different types of admissions, Ontario, 2007-2011**

| **Most responsible diagnosis** | **N** | **Amputation**  **%** | **Debridement**  **%** | **Dressing**  **%** | **Bypass leg**  **%** | **Angioplasty**  **%** | **Drainage**  **%** | **Procurement**  **%** | **Antibiotics**  **%** |
| --- | --- | --- | --- | --- | --- | --- | --- | --- | --- |
| DFU | 4,040 | 19.55 | 18.81 | 5.50 | 2.75 | 2.45 | 1.41 | 0.59 | 0.15 |
| DFU with gangrene | 4,179 | 61.45 | 19.07 | 5.72 | 4.93 | 4.26 | 0.72 | 0.67 | 0.12 |
| Infection/sepsis | 3,294 | 4.58 | 4.10 | 0.97 | 0.03 | 0.33 | 0.30 | 0.09 | 0.30 |
| Cellulitis lower limb | 2,885 | 1.80 | 5.30 | 1.25 | 0.07 | 0.28 | 1.04 | 0.14 | 0.24 |
| Ulcer lower limb | 712 | 80.48 | 18.26 | 4.92 | 1.26 | 1.26 | 0.70 | 1.83 | 0.42 |
| Osteomyelitis | 1,424 | 30.55 | 21.63 | 3.02 | 0.28 | 0.77 | 0.77 | 0.49 | 0.00 |
| Complications procedure | 1,145 | 9.87 | 12.93 | 4.63 | 0.87 | 0.87 | 1.14 | 1.14 | 0.17 |
| Complication amputation | 633 | 59.08 | 19.43 | 6.00 | 0.32 | 0.16 | 0.79 | 1.58 | 0.00 |
| Rehabilitation | 617 | 1.78 | 1.62 | 0.16 | 0.16 | 0.00 | 0.16 | 0.00 | 0.32 |
| Palliative care | 962 | 1.25 | 0.73 | 0.10 | 0.00 | 0.10 | 0.10 | 0.00 | 0.00 |
| Convalescence | 1,245 | 0.24 | 0.88 | 0.16 | 0.00 | 0.00 | 0.08 | 0.08 | 0.40 |
| Peripheral angiopathy | 6,816 | 52.05 | 9.83 | 2.77 | 19.42 | 7.44 | 0.29 | 0.41 | 0.01 |
| Peripheral tissue problem | 2,378 | 42.68 | 42.89 | 6.14 | 4.67 | 1.89 | 0.93 | 3.15 | 0.00 |
| **Overall** | **30,330** | **42.63** | **16.81** | **3.99** | **6.06** | **3.05** | **0.68** | **0.75** | **0.11** |

DFU: Diabetic Foot Ulcer

**Additional file 1: Table S5: Frequency of interventions associated with different types of ER and clinic visits, day procedures, Ontario, 2007-2011**

| **Most responsible diagnosis** | **N** | **Amputation**  **%** | **Debridement**  **%** | **Dressing**  **%** | **Bypass leg**  **%** | **Angioplasty**  **%** | **Drainage**  **%** | **Procurement**  **%** | **Antibiotics**  **%** |
| --- | --- | --- | --- | --- | --- | --- | --- | --- | --- |
| DFU | 15,431 | 1.06 | 4.62 | 9.31 | 0.00 | 0.09 | 0.72 | 0.09 | 6.45 |
| DFU with gangrene | 2,716 | 8.98 | 5.41 | 3.98 | 0.00 | 0.48 | 0.59 | 0.07 | 5.34 |
| Infection/sepsis | 2,391 | 0.08 | 0.38 | 0.33 | 0.00 | 0.00 | 0.08 | 0.00 | 9.49 |
| Cellulitis lower limb | 16,692 | 0.05 | 0.35 | 2.54 | 0.00 | 0.00 | 0.62 | 0.00 | 13.07 |
| Ulcer lower limb | 3,489 | 0.95 | 5.30 | 8.23 | 0.00 | 0.09 | 0.43 | 0.00 | 5.73 |
| Osteomyelitis | 1,739 | 11.04 | 7.65 | 2.42 | 0.00 | 0.00 | 0.58 | 0.00 | 7.94 |
| Complications procedure | 2,882 | 0.62 | 3.16 | 2.71 | 0.03 | 0.00 | 0.45 | 0.10 | 3.47 |
| Complication amputation | 529 | 6.81 | 11.91 | 3.40 | 0.00 | 0.00 | 0.95 | 0.76 | 3.21 |
| Rehabilitation | 1 | 0.00 | 0.00 | 0.00 | 0.00 | 0.00 | 0.00 | 0.00 | 0.00 |
| Palliative care | 99 | 0.00 | 0.00 | 0.00 | 0.00 | 0.00 | 0.00 | 0.00 | 1.01 |
| Convalescence | 23 | 0.00 | 0.00 | 0.00 | 0.00 | 0.00 | 0.00 | 0.00 | 0.00 |
| Peripheral angiopathy | 4,306 | 8.15 | 3.48 | 1.51 | 0.02 | 5.74 | 0.12 | 0.02 | 2.95 |
| Peripheral tissue problem | 2,072 | 8.59 | 42.76 | 6.03 | 0.05 | 0.10 | 0.87 | 2.12 | 3.38 |
| **Overall** | **52,370** | **3.62** | **6.51** | **4.97** | **0.01** | **0.52** | **0.56** | **0.16** | **7.81** |

DFU: Diabetic Foot Ulcer

**Additional file 1: Table S6: Interventions related to DFU code, by sex, Canada, 2011**

| **Intervention** | **Women** | **Men** | **All** |
| --- | --- | --- | --- |
| Angioplasty leg | 548 | 1002 | 1,550 |
| Bypass leg (fem-pop) | 328 | 705 | 1,033 |
| Dressing | 1,414 | 3,231 | 4,645 |
| Antibiotics (intravenous or injection) | 2,261 | 4,402 | 6,663 |
| Drainage | 177 | 345 | 522 |
| Procurement | 77 | 171 | 248 |
| Debridement | 1,712 | 4,084 | 5,796 |
| Amputation: | 1,711 | 4,325 | 6,036 |
| *Femur* | *388* | *555* | *943* |
| *Knee* | *17* | *25* | *42* |
| *Tibia fibula* | *559* | *1452* | *2,011* |
| *Tarsal metatarsal ankle** | *491* | *1560* | *2,051* |
| *Phalanx* | *267* | *672* | *939* |
| *Toe* | *116* | *338* | *454* |
| **Total** | **8,228** | **18,265** | **26,493** |

*Tarsal metatarsal ankle also referred to as fore, mid and hind regions of the foot.

DFU: Diabetic Foot Ulcer

**Additional file 1: Table S7: Average length of stay and costs for admission and ER/clinic visits related to DFU, Canada, 2011 (2011 CAD$)**

| **Most responsible diagnosis** | **Average Length**  **of Stay (days)** | **Average costs*** | **ER and clinic**  **visits*** |
| --- | --- | --- | --- |
| DFU | 16 | $14,800 | $282 |
| DFU with gangrene | 24 | $24,179 | $493 |
| Infection/sepsis | 21 | $30,644 | $593 |
| Cellulitis lower limb | 12 | $9,864 | $220 |
| Ulcer lower limb | 20 | $17,209 | $184 |
| Osteomyelitis | 17 | $15,144 | $461 |
| Complication procedure | 18 | $21,088 | $275 |
| Complications amputate | 20 | $19,310 | $438 |
| Surgical dress |  | NA | $122 |
| Rehabilitation | 45 | $33,207 | $69 |
| Palliative care | 22 | $17,560 | $412 |
| Convalescence | 19 | $15,571 | $304 |
| Problems health care | 57 | $19,410 | $486 |
| Peripheral angiopathy | 19 | $20,257 | $785 |
| Peripheral tissue problem | 14 | $11,633 | $403 |

*Average costs represent hospital budget portion only.

DFU: Diabetic Foot Ulcer. ER: Emergency Room

**Additional file 1: Table S8: Physician fees for interventions, Ontario, (2011 CAD$)**

| **Intervention** | **Duration**  **(minutes)** | **Surgery** | **Pre surgery** | **Post-surgery** | **Total** |
| --- | --- | --- | --- | --- | --- |
| Amputation: |  |  |  |  |  |
| Above Knee | 90 | $719 | $493 | $856 | $2,067 |
| Knee | 90 | $719 | $493 | $856 | $2,067 |
| Below knee | 90 | $719 | $493 | $856 | $2,067 |
| Fore-mid-hind foot | 90 | $531 | $453 | $700 | $1,683 |
| Phalanx | 90 | $638 | $493 | $700 | $1,830 |
| Toe | 60 | $734 | $90 | $583 | $1,407 |
| Femoral artery bypass | 180 | $1,482 | $493 | $583 | $2,558 |
| Debridement | 60 | $398 | $142 | $107 | $647 |
| Cellulitis abscess and drainage | 60 | $513 | $142 | $107 | $762 |
| Procurement | 90 | $481 | $142 | $107 | $730 |
| Angioplasty | 120 | $1,536 | $493 | $106 | $2,136 |

**Additional file 1: Table S9: Baseline demographics of incident DFU cohort**

| **Characteristic** | **n (%)** |
| --- | --- |
| Cases, *n* | 5,015 |
| Age, mean (SD) (min, max) | 68 (14) (19, 102) |
| Men | 3,028 (60%) |
| Diseases arteries and capillaries | 1,435 (29%) |
| Renal Failure | 1,248 (25%) |
| Ischemic Heart Disease | 1,107 (22%) |
| Heart failure | 1,020 (20%) |
| Cancer | 594 (12%) |
| Chronic lower respiratory disease | 541 (11%) |
| Stroke or TIA | 310 (6%) |
| Hypertension | 121 (2%) |

DFU: Diabetic Foot Ulcer. SD: Standard deviation

**Additional file 1: Table S10: Incident based analysis, summary of reasons for admission or ER/clinic visit, by year, 2009-2011**

| **Admission or ER visit reason:** | **Year 1** | **% of cohort** | **Year 2** | **% of cohort** | **Year 3** | **% of cohort** |
| --- | --- | --- | --- | --- | --- | --- |
| *Cases (n)* | *5,015* | *100.0* | *2,979* | *59.3* | *2,376* | *47.3* |
| DFU | 1559 | 31.0 | 348 | 6.9 | 236 | 4.7 |
| DFU with gangrene | 622 | 12.4 | 175 | 3.5 | 97 | 1.9 |
| Infection/sepsis | 377 | 7.5 | 192 | 3.8 | 142 | 2.8 |
| Cellulitis lower limb | 1082 | 21.5 | 376 | 7.5 | 319 | 6.3 |
| Ulcer lower limb | 54 | 1.1 | 17 | 0.3 | 10 | 0.2 |
| Osteomyelitis | 206 | 4.1 | 109 | 2.2 | 71 | 1.4 |
| Complication procedure | 212 | 4.2 | 147 | 2.9 | 81 | 1.6 |
| Complications amputate | 71 | 1.4 | 41 | 0.8 | 29 | 0.6 |
| Surgical dress | 222 | 4.4 | 84 | 1.7 | 70 | 1.4 |
| Rehabilitation | 49 | 1.0 | 21 | 0.4 | 15 | 0.3 |
| Palliative care | 93 | 1.9 | 61 | 1.2 | 35 | 0.7 |
| Convalescence | 119 | 2.4 | 55 | 1.1 | 28 | 0.6 |
| Problems health care | 0 | 0.0 | 0 | 0.0 | 1 | 0.0 |
| Peripheral angiopathy | 861 | 17.1 | 312 | 6.2 | 151 | 3.0 |
| Peripheral tissue problem | 145 | 2.9 | 65 | 1.3 | 33 | 0.7 |

Index year for incidence cohort was 2009.

DFU: Diabetic Foot Ulcer. ER: Emergency Room

**Additional file 1: Table S11: Incident based analysis, types of amputations, by year, 2009-2011**

| **Amputation Type** | **Year 1** | **% of cohort** | **Year 2** | **% of cohort** | **Year 3** | **% of cohort** |
| --- | --- | --- | --- | --- | --- | --- |
| *Cases (n)* | *5,015* | *100.0* | *2,979* | *59.3* | *2,376* | *47.3* |
| Amputation (any) | 883 | 17.6 | 367 | 7.3 | 187 | 3.7 |
| Femur | 166 | 3.3 | 92 | 1.8 | 35 | 0.7 |
| Knee | 3 | 0.1 | 2 | 0.0 | 1 | 0.0 |
| Tibia fibula | 333 | 6.6 | 158 | 3.1 | 82 | 1.6 |
| Tarsal, metatarsal ankle | 293 | 5.8 | 103 | 2.0 | 52 | 1.0 |
| Phalanx | 160 | 3.2 | 61 | 1.2 | 33 | 0.7 |
| Toe | 86 | 1.7 | 32 | 0.6 | 15 | 0.3 |

Index year for incidence cohort was 2009
